# Unique Coexistence of Left Inferior Suprarenal, Accessory Renal, and Arched Testicular Arteries Arising From a Common Trunk: A Rare Vascular Variation With Dual Left Testicular Veins

**DOI:** 10.7759/cureus.105662

**Published:** 2026-03-22

**Authors:** Maxwell Rowley, Willem Northcut, Anthony A Hewetson, Keith N Bishop, Gurvinder Kaur

**Affiliations:** 1 School of Medicine, Texas Tech University Health Sciences Center, Lubbock, USA; 2 Department of Medical Education, Texas Tech University Health Sciences Center, Lubbock, USA

**Keywords:** arched testicular artery, cadaveric case report, duplicated testicular veins, inferior polar artery, inferior suprarenal artery

## Abstract

Visceral blood vessels often display significant anatomical variability, with testicular artery (TA) anomalies more frequently observed on the left side. During a routine cadaveric dissection of an 88-year-old Caucasian male with no known kidney or testicular disease, a unique vascular pattern was identified. A left common trunk arose from the abdominal aorta at approximately the level of L2 vertebra, positioned partially behind the left renal vein. This trunk ascended and bifurcated into superior and inferior branches. The superior branch gave rise to a left inferior suprarenal artery and a left inferior polar renal artery. The inferior branch looped around the left renal vein and artery, continued as an arched left TA. The TA then traveled inferiorly, penetrating the deep inguinal ring and entering the spermatic cord. Additionally, two left testicular veins were observed traversing a typical course alongside the TA contributing to the left renal vein. This case presents a rare vascular configuration in which multiple individually reported variations - the left inferior suprarenal artery, inferior polar renal artery, and arched testicular artery - not only coexist, but all arise from a common trunk. To the best of our knowledge, this precise anatomical pattern has not been previously reported in the literature. The coexistence of dual left testicular veins further highlights the complexity of this case. Awareness of these anatomical variations is pertinent to surgical specialists and interventional radiologists in reducing complications in retroperitoneal procedures as well as improving procedural accuracy.

## Introduction

The testicular arteries are paired vessels that typically originate from the anterolateral aspect of the abdominal aorta around the level of the second lumbar vertebra. Each artery descends along the posterior abdominal wall, then approaches and passes through the deep inguinal ring to become a component of the spermatic cord [1]. Numerous published studies have described a wide range of anatomical variations in both the site of origin and the subsequent courses of these arteries. For instance, the testicular arteries may also originate from the renal artery, middle suprarenal artery, and lumbar artery. Rarely it can arise from the common or internal iliac arteries, or from the inferior epigastric artery. Also, they may arise from a common trunk or be doubled, tripled, or quadrupled [2-13]. In the abdomen, the testicular artery supplies the perirenal fat, ureter, and external iliac lymph nodes; in the inguinal canal, it supplies the cremaster muscle [2-13]. The presence of an arched testicular artery (TA), also known as the arched testicular artery of Luschka, coursing over the renal vein has been documented [5,7,11,14,15-17]. Variations in the testicular artery, including its arched form or the presence of branches supplying the suprarenal gland, have been similarly described [3,4,8,16,18]. Furthermore, the origin of renal parenchymal branches from the testicular artery has been reported [19]. To the best of our knowledge, this is the first documented case of a common trunk bifurcating into superior and inferior branches. The inferior branch of the common trunk arched over the renal vessels and continued as the left arched TA. Additionally, two left testicular veins followed the typical course alongside the TA and drained into the left renal vein.

## Case presentation

The study involved a routine cadaveric abdominal dissection in an institutional anatomy lab of an 88-year-old Caucasian male cadaver with unremarkable renal or testicular findings. The underlying cause of death was determined to be attributed to severe protein malnutrition with essential hypertension. The cadaver was embalmed with a proprietary fluid containing ethanol, formaldehyde, ethylene glycol, phenol, and glutaraldehyde obtained from a certified supplier. Forty-eight ounces of the solution were diluted in 3 gallons of water and injected into the vascular system, repeated 2-3 times based on body weight and fluid distribution, using a guideline of 1 gallon per 50 pounds. After injection, the cadaver was immersed in a 10% phenol bath for 24 hours. The testicular arteries were examined and photographed to document their origins and courses.

**Figure 1:**
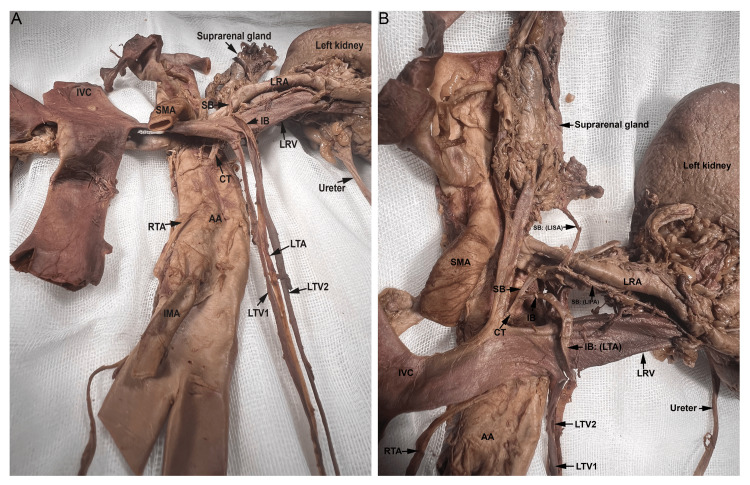
Arched left testicular artery. (A) The common trunk (CT) shown from the inferior view with the left renal vein (LRV) lifted. The CT gave rise to the left testicular artery (LTA), left inferior suprarenal artery (LISA), and left inferior polar renal artery (LIPA) and was covered by the LRV. (B) Photographs taken during dissection show that the CT gave rise to two branches, superior branch (SB) and inferior branch (IB). The SB further bifurcated into the LISA and the LIPA. The IB arched over the LRV and continued as the LTA. AA: Abdominal Aorta; IMA: Inferior Mesenteric Artery; IVC: Inferior Vena Cava; LRA: Left Renal Artery; LTV 1: Left Testicular Vein 1; LTV 2: Left Testicular Vein 2; RTA: Right Testicular Artery; SMA: Superior Mesenteric Artery; IB: Inferior Branch.

A common left trunk originated from the abdominal aorta near the L2 vertebra and was obscured by the left renal vein (Figure 1A).

**Figure 2:**
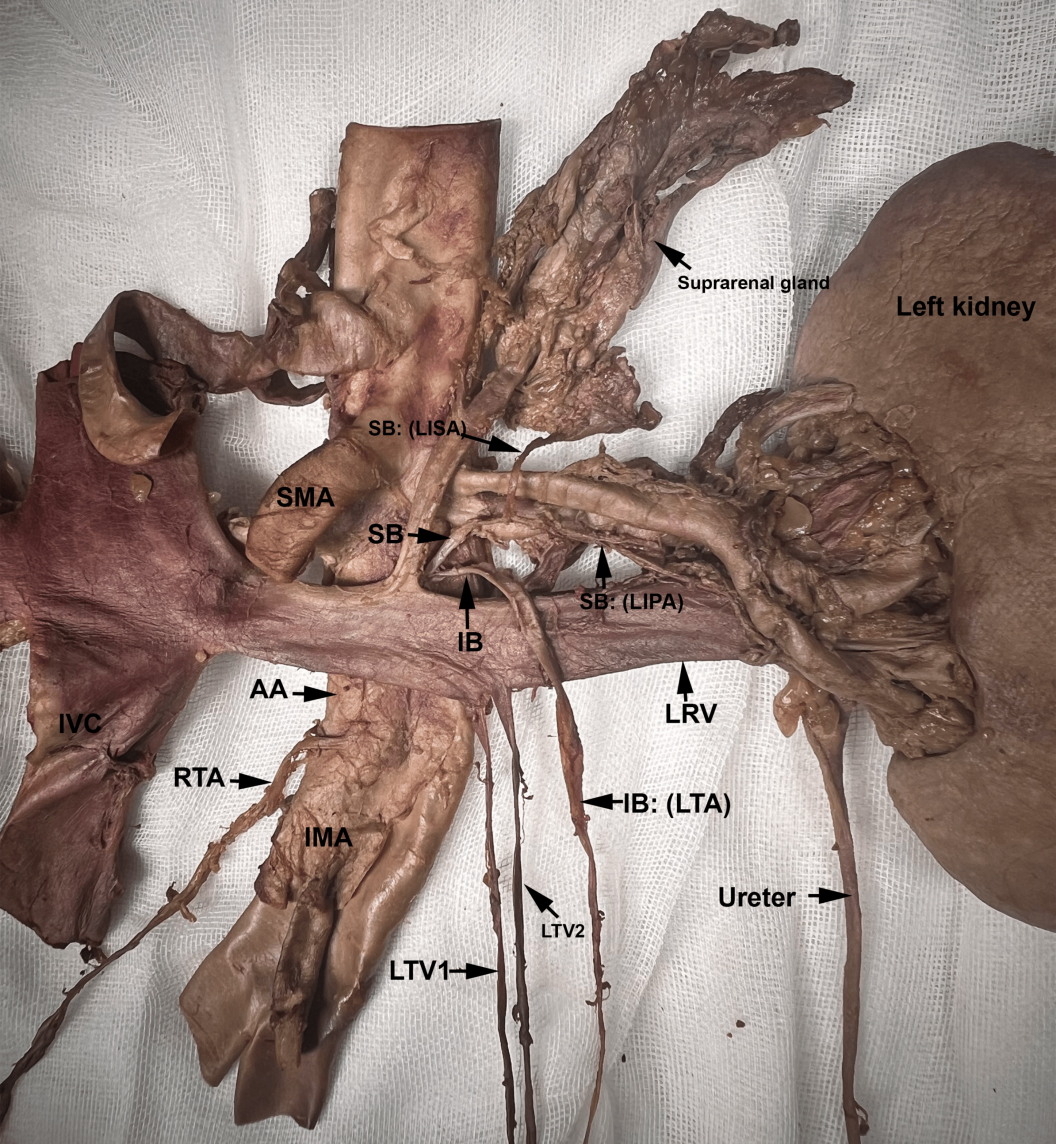
Common Trunk Giving the Superior and Inferior Branches. The zoomed-in picture taken during dissection shows the superior branch (SB) and inferior branches (IB) of the common trunk. The SB further bifurcated into the left inferior suprarenal artery (LISA) and the left inferior polar renal artery (LIPA). The IB arched over the left renal vein (LRV) and continued as the left testicular artery (LTA). AA: Abdominal Aorta; IMA: Inferior Mesenteric Artery; IVC: Inferior Vena Cava; LTV 1: Left Testicular Vein 1; LTV 2: Left Testicular Vein 2; RTA: Right Testicular Artery.

It ascended superiorly, before bifurcating into the superior and inferior branches. To the best of our knowledge, this is the first documented case of a common trunk bifurcating into superior and inferior branches. The superior branch gave rise to both a left inferior suprarenal and a left inferior polar artery. Typically, the inferior suprarenal artery is normally found originating from the renal artery and travels superiorly into the inferior aspect of the suprarenal gland. The inferior branch of the common trunk looped around the left renal vein and artery and continued as the arched left TA (Figure 1B and Figure 2). The left renal artery showed an orthotopic position and trajectory. The two left testicular veins drained into the left renal vein (Figure 2).

**Figure 3:**
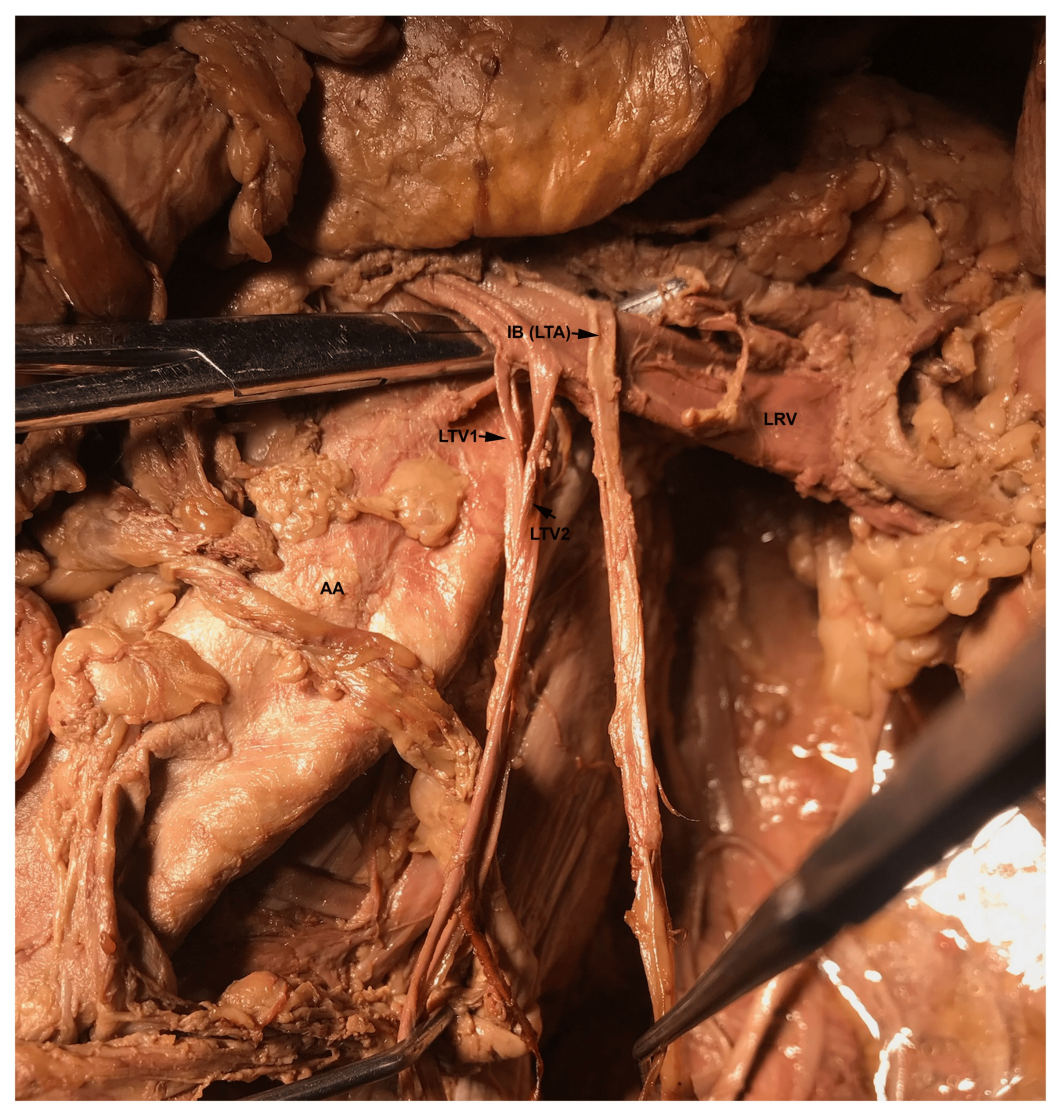
Normal Course of the Left Testicular Artery and Veins. The photograph taken during dissection shows that the left testicular artery (LTA) arched over the left renal vein (LRV). The duplicated left testicular veins (LTV) can be seen draining into the LRV. On the right side, the right testicular artery and vein followed normal origin and pattern. AA: Abdominal Aorta; IB: Inferior Branch; LTV 1: Left Testicular Vein 1; LTV 2: Left Testicular Vein 2.

The left testicular veins followed a typical course alongside the left TA (Figure 3). On the right side, the right testicular artery and vein followed normal origin and pattern.

## Discussion

The embryological development of the gonadal arteries can result in diverse origins, branching patterns, and vascular course [20, 21]. The gonadal arteries develop as lateral splanchnic branches of the aorta that enter the mesonephros. These mesonephric arteries supply the developing gonads, suprarenal glands, and diaphragm. These arteries are grouped into cranial, middle, and caudal sets, totaling nine lateral branches. Typically, as the gonads descend, one caudal mesonephric artery persists and evolves into the definitive gonadal (testicular or ovarian) artery, whereas the cranial and middle branches undergo regression. However, persistence of specific embryonic arteries can lead to anatomical variations. A high origin of the gonadal artery from the level of superior mesenteric artery or even the celiac trunk may result from the persistence of a cranial mesonephric artery [20, 21]. Similarly, a gonadal artery originating at the level of the renal pedicle is attributed to the persistence of a middle lateral mesonephric artery [20, 21]. The arched course of the gonadal artery can also be explained by its embryological development. If a mesonephric artery from the middle group - located cranial to the renal pedicle - persists, the developing gonadal artery will initially lie above the renal vessels. As the gonads descend and the kidney ascends during development, the renal vein may be positioned at a superior level than the origin of the gonadal artery. Consequently, the gonadal artery is driven to take on a curvilinear path as it loops around the renal vein [20, 21].

A total of five separate groups have attempted to classify TA variants [15-16,22-25]. Two groups classified this variation based on origin: 1) Radojevic and Stolic classified the TA variations into four groups [22], and 2) Notkovich classified the TA variations into three groups [16]. Two groups classified the TA variation based on origin and number: 3) Machnicki and Grzybiak classified the TA variations into four groups [25], and 4) Cicekcibasi et al. classified the variations into four alternative types [23]. One group classified the TA variations based on the site and level of origin: Kayalvizhi et al. classified the TA into four groups with subgroups (Table 1) [24].

**Table 1:** Summary of the Various Classifications of Testicular Artery Variants

Author, (Year, Reference)	Basis of classification	Type/Subtype	Definition/description
Radojevic & Stolic, 1964 [22]	Origin and course of the testicular artery	Type I	Most frequent pattern: TA arises from the aorta, sometimes at the same level as the renal artery
		Type II	Incidence - 5%, TA arises from the renal artery itself or from a supernumerary artery without actually arising from a true polar artery
		Type III	Incidence - 2%, TA arises from the middle suprarenal artery
		Type IV	Incidence - 2.6%, TA arises just below the renal artery to pass above and anteriorly, over the renal vein.
Notkovich, 1955 [16]	Relationship of the testicular artery to renal vein	Type I	TA descends directly without contact with the renal vein.
		Type II	TA arises from a higher level than the renal vein and crosses in front of it.
		Type III	TA arises from a lower level than the renal vein and arches around it.
Machnicki and Grzybiak, 1997 [25]	Number of arteries supplying a gonad and origin	Type A	Single TA originating from the abdominal aorta.
		Type B	Single TA originating from the renal artery.
		Type C	Two TA originating from the aorta that supplied the same gonad.
		Type D	Two TA supplying the same gonad, one arising from the aorta and the other from the renal artery.
Ciçekcibasi et al., 2002 [23]	Origin and positional level	Type I	TA originating from the suprarenal artery.
		Type II	TA originating from the renal artery.
		Type III	TA of high positional origin from the aorta, close to the renal artery lineage.
		Type IV	TA duplication originating from the aorta or from various vessels.
Kayalvizhi et al., 2017 [24]	Site and level of origin	Type I	Testicular artery arising from the abdominal aorta (four subtypes).
		Type IA	Normal origin from the aorta
		Type IB	Around renal pedicle (Corresponding, proximal, or distal to pedicle)
		Type IC	At and between superior mesenteric artery and celiac trunk
		Type ID	At distal to inferior mesenteric artery level
		Type II	Origin from a renal arterial trunk (five subtypes).
		Type IIA	From main trunk/hilar
		Type IIB	From accessory renal branch (superior/first; inferior/second and most inferior/third accessory renal arteries)
		Type IIC	From aberrant renal branch
		Type IID	From polar/capsular branch (superior or inferior)
		Type IIE	From segmental branch
		Type III	From suprarenal branch
		Type IV	Other vessels

Our observation fits well with the classification offered by Notkovich (1956), specifically Type III (Table 1) [16], often referred to as the arched testicular artery (of Luschka), which is characterized by a looping relationship to the renal vein, passing successively behind, above and then in front of it. Reported incidences for this Type III pattern vary widely in the literature, from approximately 1.7% to 22% [24]. Venous drainage from the testis begins as a network of small channels in the testis and epididymis, which coalesce to form the pampiniform plexus. This plexus surrounds the testicular artery within the spermatic cord and, as it ascends superiorly, it merges into two or three venous trunks near the deep inguinal ring. The trunks subsequently unite in the lumbar region to form a single testicular vein. The testicular vein lies anterior to the ureter and ascends adjacent to the testicular artery. On the right, it generally terminates in the inferior vena cava, whereas on the left, it most often joins the left renal vein.

A variety of deviations from this standard venous pattern have been documented, including abnormal courses, duplication of the testicular veins (potentially due to the venous testicular trunks not uniting), and unusual drainage sites into neighboring venous structures [2,26-29].

Renal arteries are a pair of lateral branches from the abdominal aorta, generally originating just below the superior mesenteric artery at the level of L1 and L2 vertebrae. The renal artery usually divides into two main trunks, which then branch into 5-7 segmental arteries that enter the renal parenchyma. In the typical pattern, each kidney is supplied by a single renal artery. The most common vascular variant is the presence of multiple renal arteries. The number of accessory renal arteries can vary considerably; between one and five have been reported. Based on their termination and the region of the kidney they supply, three accessory renal artery patterns can be distinguished: superior polar, inferior polar, and hilar arteries. These accessory arteries may arise from the abdominal aorta or from the main renal artery, and more rarely from other vessels such as the common iliac artery or the celiac trunk [30]. Likewise, numerous studies have documented variant origins of the suprarenal arteries [3,4,12]. While the individual vascular variations reported here have been documented separately in the literature, the concurrent occurrence of a left inferior suprarenal artery, a left inferior polar renal artery, and an arched left testicular artery arising from a common trunk - along with double left testicular veins draining to the left renal vein - has not been previously reported in the same individual.

Clinical awareness of vascular anomalies, such as an arched testicular artery, is crucial to prevent complications during renal or testicular interventions. The arched testicular artery could be an additional contributing factor to left renal vein hypertension. Compression of the left renal vein between the aorta and the superior mesenteric artery, commonly known as nutcracker syndrome, can obstruct venous outflow, leading to venous hypertension and/or gonadal vein reflux [31].

Aberrant gonadal vessels may potentially restrict blood flow to the kidney or gonadal glands, leading to pathological conditions such as a varicocele [15]. An understanding of renal vascular anomalies is especially important for clinicians involved in kidney retrieval and transplantation, endourologic procedures, and various interventional techniques. Percutaneous renal biopsy, a procedure in which kidney tissue is sampled using ultrasound-guided needles, is particularly susceptible to complications when aberrant vasculature is present. In these settings, unrecognized vascular anomalies can increase the risk of retroperitoneal hemorrhage. Ahmed et al. reported a rare case involving dual arterial injury during a percutaneous renal biopsy [32]. Both an accessory renal artery and an aberrant left testicular artery were inadvertently punctured, leading to a substantial hematoma. Further evaluation revealed that the left testicular artery originated atypically from a capsular artery, rather than its normal origin. This case, along with similar anatomical variations identified in our findings, highlights the importance of pre-procedural imaging to identify vascular anomalies. Diagnostic tools such as angiography and 3D CT angiography can provide critical insights into vascular architecture, potentially reducing surgical errors and enhancing patient safety. The unique vascular pattern described in this case report not only underscores the anatomical variability of the gonadal vasculature but also emphasizes the importance of recognizing such variants in surgical and radiological contexts.

## Conclusions

This case highlights a rare and unique constellation of vascular variations involving the gonadal and renal vasculature. Based on our review, the finding of a common arterial trunk bifurcating into a superior branch - giving rise to the left inferior suprarenal artery and a left inferior polar renal artery - and an inferior branch continuing as an arched left testicular artery coursing over the renal vein has not been previously reported. Additionally, the occurrence of two ipsilateral left testicular veins draining into the left renal vein further adds to the complexity of this anatomical configuration. Such vascular variations, although individually reported, have not been observed concurrently in a single individual. Understanding these anomalies is critical for radiologists, urologists, and surgeons, especially in the context of renal transplantation, varicocelectomy, and other retroperitoneal interventions. Awareness of these patterns can prevent inadvertent vascular injury, improve surgical planning, and minimize postoperative complications.
